# Is normal-tension glaucoma a risk factor for stroke?—A 10-year follow-up study

**DOI:** 10.1371/journal.pone.0179307

**Published:** 2017-06-19

**Authors:** Meng-Sheng Lee, Li-Lin Kuo, Elise Chia-Hui Tan, Oscar K. Lee

**Affiliations:** 1 Department of Ophthalmology, Taipei City Hospital, Taipei, Taiwan; 2 Zhongxiao Branch, Taipei City Hospital, Taipei, Taiwan; 3 Institute of Public Health and Community Medicine Research Center, National Yang-Ming University, Taipei, Taiwan; 4 Institute of Health Policy and Management, College of Public Health, National Taiwan University, Taipei, Taiwan; 5 National Research Institute of Chinese Medicine, Ministry of Health and Welfare, Taipei, Taiwan; 6 Institute of Clinical Medicine, National Yang-Ming University, Taipei, Taiwan; 7 Department of Education and Research, Taipei City Hospital, Taipei, Taiwan; Soochow University Medical College, CHINA

## Abstract

**Objectives:**

To investigate whether patients with normal-tension glaucoma (NTG) have a higher incidence of stroke.

**Design:**

A population-based retrospective cohort study based on data from the Taiwan National Health Insurance Research Database (NHIRD) from January 1, 2001, to December 31, 2010.

**Methods:**

Data were retrospectively collected from the NHIRD. A total of 245 (20.1%) patients with a history of stroke at the time of glaucoma diagnosis were excluded, and 1,218 patients with NTG who were 20 years of age and older were identified. Patients’ age, gender and pre-existing comorbidities, including hypertension, diabetes, congestive heart failure, ischemic heart disease, atrial fibrillation and disorders of lipid metabolism, were recorded. The propensity score method with a 1:5 matching ratio was used to minimize selection bias. Cox regression with robust variance estimation was used to estimate the hazard ratio (HR) of developing stroke between the NTG and control groups.

**Results:**

After adjusting for patient age, gender, and pre-existing comorbidities, the HR was 6.34, indicating that the incidence of stroke was significantly higher in patients with NTG than in controls. Furthermore, a higher risk of stroke was also found in most subgroups with the above-mentioned comorbidities.

**Conclusion:**

NTG is a significant risk factor for subsequent stroke in most of the described comorbidity subgroups. Early interventions for stroke prevention should be provided to newly diagnosed patients with NTG.

## Introduction

Glaucoma refers to a group of eye diseases characterized by optic neuropathy that is often accompanied by elevated intraocular pressure (IOP), which may result in irreversible optic nerve damage and blindness if not treated adequately. Glaucoma is one of the leading causes of blindness following cataracts [1–5]. Primary open-angle glaucoma (POAG) is the most prevalent form of glaucoma. Most patients with POAG are asymptomatic or have only very mild nonspecific symptoms in the early to mid-stages of disease before developing advanced visual function disturbances [6–10]. Normal-tension glaucoma (NTG) is a unique form of glaucoma similar to POAG that can lead to glaucomatous disc atrophy and occasionally disc hemorrhage with typical visual field defects, but IOP elevation does not occur [11].

Like most chronic forms of glaucoma, symptoms do not manifest early in NTG. In particular, NTG cannot be diagnosed simply by IOP measurement. NTG is considered a multifactorial disease, and vascular risk factors may play an important role in its etiology [12]. Patients with NTG are reported to suffer from vasospastic conditions such as migraine [13], Raynaud’s phenomenon [14], blood flow disturbances such as hemodynamic crisis, low systemic blood pressure, nocturnal dips in blood pressure [15], hypertension (HTN) [15,16], and changes in ciliary circulation [12,15,17,18].

The pathophysiology of NTG remains poorly understood. Ocular and systemic circulation abnormalities have been linked to the cause and progression of both POAG [19–24] and NTG [25–28]. However, there are structural [29,30] and functional [31–35] differences between POAG and NTG, and it is postulated that similar to ischemic stroke, NTG may also be due to a vascular perfusion problem.

While there is scattered evidence in the literature suggesting a correlation between NTG and vascular diseases, particularly stroke, there is a lack of large-scale studies attempting to directly elucidate a causal relationship between NTG and stroke. The aim of this study was to compare the incidence of stroke between patients with NTG and individuals in a matched comparison group using population-based claims data. Patients diagnosed with NTG were hypothesized to possess a greater risk of developing stroke.

## Methods

### Database and study population

Data were collected from the National Health Insurance Research Database (NHIRD), which was released by the Taiwan National Health Research Institute. The National Health Insurance (NHI), a single-payer covering more than 99% of Taiwan’s 23 million residents, was implemented in 1995. In the NHIRD, all of the identification information is scrambled. It is a longitudinal, individual-based database containing all medical claims data for one million enrollees who are randomly sampled from the NHI registry. The NHI enrollees included in the NHIRD do not differ significantly in age, gender, or overall healthcare costs from the overall population of NHI enrollees. Therefore, the NHIRD provides a unique resource with which to examine the relationship between NTG and stroke. The data used in this study were obtained from the NHI claims data of one million enrollees collected between 2001 and 2010.

A retrospective cohort study was then conducted using the NHIRD. In this study, patients with NTG were identified from the NHIRD between January 1, 2001, and December 31, 2010. Patients diagnosed with NTG (ICD-9-CM: 365.12) on three or more ambulatory care claims or in an inpatient setting were eligible to ensure the validity and reliability of the diagnosis. Because NTG and stroke occur less commonly in children and adolescents, the age range was restricted to patients over 20 years old. Using these methods, 1,218 NTG patients greater than 20 years old were identified. A total of 245 patients who had a history of stroke (20.1%) were excluded.

Patient age, gender and pre-exiting comorbidities, including HTN (ICD-9-CM: 401–405), diabetes (ICD-9-CM: 250), congestive heart failure (CHF) (ICD-9-CM: 428), ischemic heart disease (IHD) (ICD-9-CM: 410–414), atrial fibrillation (AF) (ICD-9-CM: 427) and disorders of lipid metabolism (ICD-9-CM: 272), were gathered from the NHI claims data.

The comparison group was extracted from the remaining patients in the NHIRD. We excluded patients who were diagnosed with any other type of glaucoma or who were diagnosed with stroke. Each patient with NTG was assigned an index date of diagnosis, which was defined as the date of the first recording of NTG, and a matched control was assigned with an identical mock index date of diagnosis [36,37].

### Ethics standard

This study was approved by the ethics board of Taipei City Hospital (approval number: 1021204-E). All participants’ identifying information was scrambled in the NHIRD.

### Outcomes

The primary outcome of the study was the occurrence of an episode of stroke (ICD-9-CM: 430–438), which was defined as the occurrence of a visit to an emergency department or admission to a hospital for stroke after the index date.

### Statistical analysis

The propensity score method (PSM) was employed to minimize selection bias and avoid problems of endogeneity [38]. The propensity score was used to transform all matching variables to a conditional probability to balance the covariate between the treatment and the comparison group to approach a random distribution and reduce the impact of covariates on the results [39,40]. Using a logistic regression model, we determined a propensity score for a NTG or non-NTG diagnosis within the study period. The covariates entered into the propensity score were personal characteristics (age, gender) and comorbidities (HTN, diabetes, CHF, IHD, AF, and disorders of lipid metabolism). We matched the NTG group with the comparison group on the logit of the propensity score, using calipers of width equal to 0.2 of the standard deviation of the logit of the propensity score. A matching ratio of 1:5 was used.

After propensity matching, a total of 5,658 cohort participants were included in the study, 943 in the NTG group and 4,715 in the comparison group. Thirty patients with NTG were excluded due to incomplete matching to 5 controls. There was no significant difference in age, gender, HTN, diabetes, CHF, IHD, AF, or disorders of lipid metabolism between the two groups. All subsequent analyses were performed in the matched samples by methods appropriate for the analysis of matched data in estimating the treatment effect and the statistical significance. Kaplan-Meier survival analyses were performed in both groups with an end point of the first stroke event, and we compared the time to outcome using a stratified log-rank test [41]. The hazard ratio (HR) for a stroke event occurrence was determined using Cox proportional hazard regression with robust variance estimation [42].

Analyses of the six subgroups stratified by different comorbidities were performed. All the above-mentioned statistical analyses were performed using SAS version 9.3 for Windows (SAS Institute, Cary, NC, USA).

## Results

**Fig 1:**
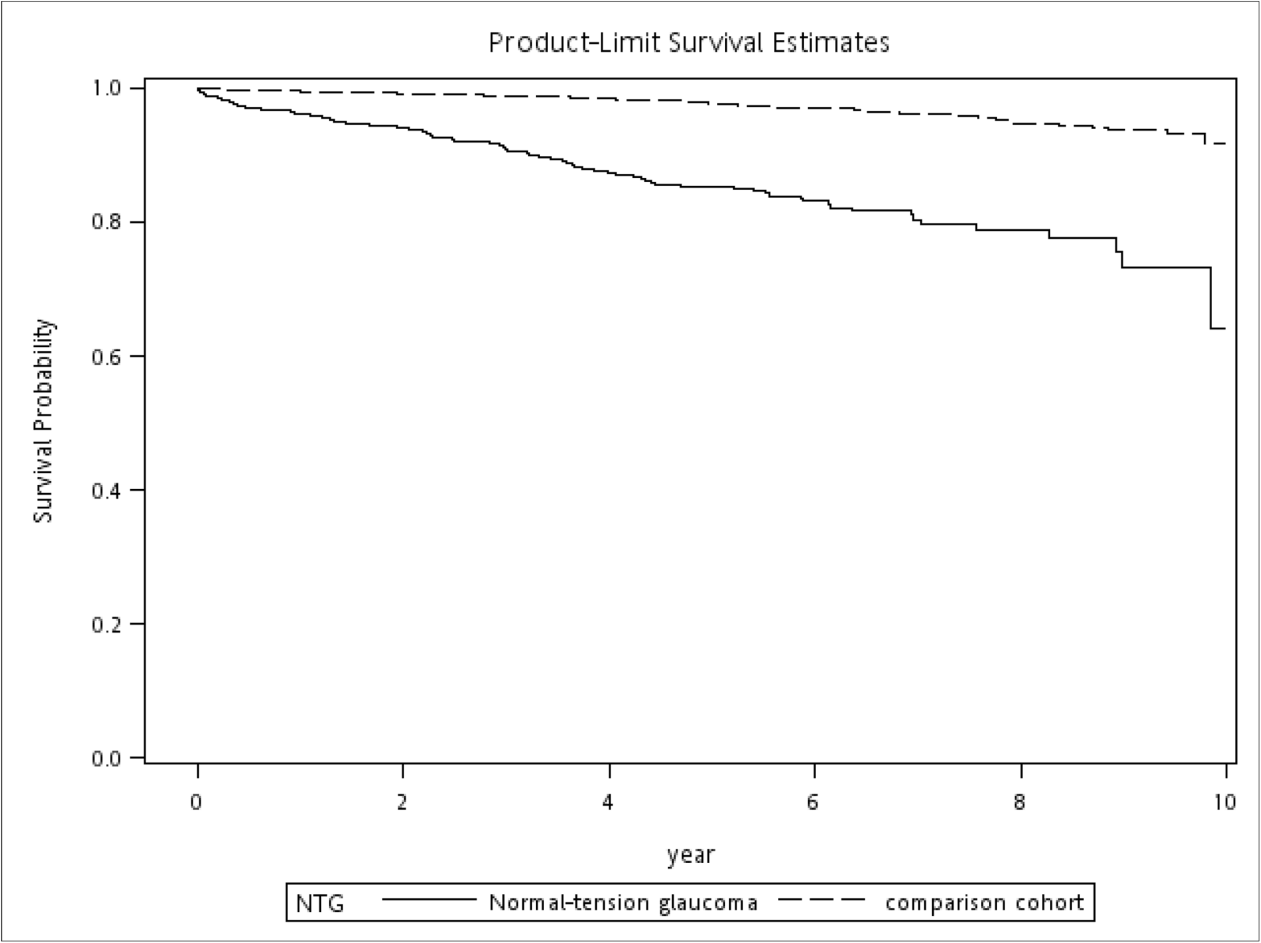
Stroke-free survival rates for NTG patients and comparison group in Taiwan, 2001 to 2010. The stratified log-rank test also indicated that patients with NTG had significantly lower stroke-free survival rates than comparison group (*P* < .0001).

The distribution of demographic characteristics and selected comorbidities for these two cohorts are presented in Table 1. The mean age was 53.84 ± 16.11 years among patients with NTG and 54.35 ± 16.27 years in the comparison group. A total of 48.57% of the sampled patients were female. In the total sample of 5,658 patients, 203 (3.59%) developed stroke during the 10-year follow-up period, including 107 in the NTG group (11.35%) and 96 in the comparison group (2.04%). The crude HR for patients with NTG was 6.20 (95% confidence interval, 4.71–8.16; *P*<0.0001). The stratified log-rank test also indicated that patients with NTG had significantly lower stroke-free survival rates (*P*<0.0001) (Fig 1).

**Table 1:** Demographic characteristics and comorbid medical disorders for patients with NTG and patients in a comparison group in Taiwan, 2001–2010 (N = 5,658).

Variable	NTG group *(n* = 943)		Comparison group *(n* = 4,715)		*P* Value
	n	%	n	%	
**Gender**					
Female	458	48.57	2,290	48.57	1.0000
Male	485	51.43	2,425	51.43	
**Age**, years, mean (SD)	53.84 (16.11)		54.35 (16.27)		0.3842
**Comorbidities**					
Hypertension					
No	739	78.37	3,645	77.31	0.4946
Yes	204	21.63	1070	22.69	
Diabetes					
No	832	88.23	4,150	88.02	0.9124
Yes	111	11.77	565	11.98	
Congestive heart failure					
No	935	99.15	4,649	98.6	0.2092
Yes	8	0.85	66	1.4	
Ischemic heart disease					
No	878	93.11	4,391	93.13	1.0000
Yes	65	6.89	324	6.87	
Atrial fibrillation					
No	912	96.71	4,553	96.56	0.9217
Yes	31	3.29	162	3.44	
Disorders of lipid metabolism					
No	844	89.5	4,140	87.8	0.1522
Yes	99	10.5	575	12.2	

After adjustment for patient age, gender, HTN, diabetes, CHF, IHD, AF, and disorders of lipid metabolism, the HR was 6.34-fold greater in patients with NTG (95% CI, 4.80–8.38; *P*<0.0001), as shown in Table 2.

**Table 2:** Stroke occurrence among patients with NTG and patients in the comparison group in Taiwan during 2001–2010 (N = 5,658).

	Stroke Occurrence					
	Yes, n (%)		No, n (%)		Crude HR (95% CI)	Adjusted HR^a^ (95% CI)
**NTG**	107	(11.35)	836	(88.65)	6.20 (4.71–8.16) ^b^	6.34 (4.80–8.38) ^b^
**Comparison**	96	(2.04)	4,619	(97.96)	1.00	1.00

^a^ Adjustment for gender, age, hypertension, diabetes, congestive heart failure, ischemic heart disease, atrial fibrillation, disorders of lipid metabolism.

^b^ *P*<0.0001

The results of the subgroup analysis stratified by different comorbidities are shown in Table 3. After adjustment for HTN, the HR for stroke was 5.35 (95% CI, 3.44–8.32; *P*<0.0001) in patients with NTG. After adjustment for diabetes, the HR for stroke was 6.88 (95% CI, 3.62–13.05; *P*<0.0001) in patients with NTG. After adjustment for CHF, the HR for stroke was 5.32 (95% CI, 0.20–140.41, *P* = 0.2362) in patients with NTG. After adjustment for IHD, the HR for stroke was 5.29 (95% CI, 2.49–11.27, *P*<0.0001) in patients with NTG. After adjustment for AF, the HR for stroke was 4.64 (95% CI, 1.57–13.71; *P*<0.0001) in patients with NTG. After adjustment for disorders of lipid metabolism, the HR for stroke was 4.38 (95% CI, 2.08–9.22; *P*<0.0001) in patients with NTG.

**Table 3:** Adjusted HR for stroke during the follow-up period for patients with NTG as compared with patients in the comparison group, stratified by different comorbidities.

	Adjusted HR^a^ (95% CI)		*P* Value
**Hypertension (yes)**	5.35	(3.44–8.32)	<0.0001
**Diabetes (yes)**	6.88	(3.62–13.05)	<0.0001
**Congestive heart failure (yes)**	5.32	(0.20–140.41)	0.2362
**Ischemic heart disease (yes)**	5.29	(2.49–11.27)	<0.0001
**Atrial fibrillation (yes)**	4.64	(1.57–13.71)	<0.0001
**Disorders of lipid metabolism (yes)**	4.38	(2.08–9.22)	<0.0001

^a^ Adjustment for gender, age and comorbidities.

## Discussion

In this retrospective cohort study, we demonstrated that NTG significantly increases the risk of stroke after adjustment for age, gender and medical comorbidities related to cerebrovascular diseases. This is, to the best of our knowledge, the first study to report a correlation between NTG and the subsequent development of stroke.

By traditional definition, an IOP of 21 mmHg was used as a cut-off diagnosis value for glaucoma. Glaucoma can actually develop at any IOP level, with typical glaucomatous optic nerve head damage and visual field changes [17,43], but the underlying cause remains unknown. Controversies exist regarding whether NTG represents a variation of POAG or a different disease entity. It is believed that NTG is more IOP-independent than POAG. IOP reduction in patients with NTG does not exert a beneficial effect on disease progression as it does for patients with POAG [44–47]. Recent studies have suggested that POAG is a disease continuum extending from a predominantly IOP-dependent entity (pure POAG) to a predominantly IOP-independent entity (pure NTG) [48–50]. Additionally, it has been proposed that the onset and progression of both forms of glaucoma, POAG, and NTG, are dictated by a degree of interaction between IOP and vascular factors [51].

Vascular insufficiency in the central nervous system has been related to the pathogenesis of NTG [8]. Cerebrovascular diseases are more frequent in patients with NTG compared with patients with high-tension glaucoma or without glaucoma [52].

The incidence of a glaucomatous optic disk appearance is significantly higher in patients with symptomatic atherosclerotic cerebrovascular diseases, and it leads to NTG [53]. In addition, the presence of a silent cerebral infarct is reported to be an independent risk factor for visual field progression in patients with newly diagnosed NTG [54]. Previous studies have shown that cerebrovascular diseases are associated with NTG. Ischemic changes in brain magnetic resonance imaging are more common in these patients [17,55]. Optic disc hemorrhage, a strong indicator of glaucomatous optic disc damage in NTG, has been reported to be associated with HTN and diabetes, which are also risk factors associated with ischemic stroke [56]. HTN, diabetes, and hypercholesterolemia are well-known risk factors for atherosclerosis and stroke [57]. Direct microvascular damage from systemic hypertension could impair blood flow to the optic disc [58,59], causing hypoxia. Prolonged hyperglycemia and lipid anomalies may increase the risk of neuronal injury from stress [60], which is regulated by nitric oxide synthase [61,62]. Other studies have shown decreased retinal blood flow in diabetic eyes due to poor function of auto-regulation [63]. At a molecular level, hypoxia-inducible factor 1-alpha is increased in the retina and optic nerve head of glaucomatous donor eyes, supporting the presence of tissue hypoxia in glaucoma [64].

In this study, we used a population-based dataset that covers more than 99% of Taiwan’s 23 million residents. Within the dataset, PSM was used to match all the variables to minimize selection bias while preserving an adequate sample size. The diagnosis of NTG was made by board-certified ophthalmologists once the patients experienced glaucomatous disc atrophy, occasional disc hemorrhage, and the typical visual field defects without documented IOP elevation. Compared with the comparison group, the adjusted HR of stroke occurrence among the NTG group (ICD-9-CM: 365.12) was 6.34, which is much higher than the value reported in a previous study regarding the risk of stroke associated with open-angle glaucoma (OAG) (ICD-9-CM: 365.1 to 365.11) [65]. This discrepancy may be due to a higher prevalence of vascular insufficiency in NTG than in OAG. In addition, we also found that the average age of patients with NTG in this study was 53.84 years, which was much younger than the average age of 60.2 years reported in the OAG study [65].

In addition, we found that NTG is a strong indicator of future stroke after adjustment for the diagnosis of comorbidities, including HTN, diabetes, IHD, AF, and disorders of lipid metabolism. There was no significant difference in the subgroup of NTG with CHF due to a small sample size (n = 74).

As a “silent vision killer,” glaucoma is not well understood in the general population. IOP is not always evaluated in adult wellness examinations even though it is known that high IOP is an important risk factor for blindness. In addition, the possibility of NTG cannot be ruled out simply by IOP screening tests. Complete fundus and visual field examinations are needed for the definitive diagnosis of NTG. We recommend a good referral system and cooperation between physicians and ophthalmologists for patients with any systemic cardiovascular diseases and other comorbidities as described.

The limitations of the study are as follows. The prevalence of NTG may have been underestimated due to the use of a secondary claims database. Nevertheless, the NHI program covers almost 99% of the residents in Taiwan and requires ophthalmologists to present evidence of their diagnosis, including visual field defects and fundus exams, to obtain approval for medications to treat patients with NTG. Thus, those who were diagnosed with NTG were bona fide patients. Another limitation is that the claims data did not include details on the severity of NTG, and thus we could not correlate the incidence of stroke with the severity of NTG. For the same reason, although low systolic blood pressure is a risk factor for NTG [15], we could not obtain blood pressure data from the NHI claims data. Patients with an IOP less than 21 mmHg were diagnosed with NTG; however, their exact IOP values are not available from the claims data. Therefore, we were unable to analyze the effect of diurnal IOP changes in patients with NTG. In addition, data regarding systolic and diastolic blood pressure were not available. The inclusion of hypertension in this model may not be adequately adjusted for the confounding effects of low systemic blood pressure.

In summary, patients with NTG, which is more difficult to diagnose than OAG, harbor a significantly higher risk of developing stroke based on this case-control longitudinal study, and the result was unchanged after adjustment for most chronic comorbidities. For patients with NTG, we recommend education regarding chronic comorbidities. Referral of patients with NTG to physicians to identify possible causes or chronic comorbidities, such as HTN, diabetes, disorders of lipid metabolism, and cardiovascular diseases, including carotid artery lesions, should be performed. Early intervention, such as the administration of antihypertensive agents, oral hypoglycemic agents, insulin, lipid-lowering agents, or even prophylactic antiplatelet agents, should also be provided to prevent the development of atherosclerotic stenosis and thrombosis and to reduce the risk of stroke. Most importantly, physicians should pay close attention to patients with the above-described chronic comorbidities and refer them to an ophthalmologist for NTG screening to prevent loss of vision and life-threatening stroke.
